# Management of Risk Factors Associated with Chronic Oral Lesions in Sheep

**DOI:** 10.3390/ani10091529

**Published:** 2020-08-30

**Authors:** Marta Ruiz de Arcaute, Delia Lacasta, José María González, Luis Miguel Ferrer, Miren Ortega, Héctor Ruiz, José Antonio Ventura, Juan José Ramos

**Affiliations:** 1 Animal Pathology Department, Instituto Agroalimentario de Aragón-IA2 (Universidad de Zaragoza-CITA), Veterinary Faculty of Zaragoza, C/Miguel Servet 177, 50013 Zaragoza, Spain; martarda@unizar.es (M.R.d.A.); jmgsovino@gmail.com (J.M.G.); lmferrer@unizar.es (L.M.F.); emille7@hotmail.com (M.O.); hectorruiz353@gmail.com (H.R.); jovepra@gmail.com (J.A.V.); jjramos@unizar.es (J.J.R.); 2 Gabinete Técnico Veterinario S.L, C/Isla conejera, sn, 50013 Zaragoza, Spain

**Keywords:** sheep, mandibular disorders, maize straw, silage, mineral supplementation

## Abstract

**Simple Summary:**

Oral injuries are widespread disorders of sheep that can cause significant economic losses in farms. These usually painful conditions prevent animals from feeding properly, leading to chronic weight loss and, very often, causing the final culling of the animals. This study analyses the management risk factors associated with the presence of mandibular and maxillary disorders in a representative cohort of sheep in Spain. These lesions are usually the external and final manifestation of underdiagnosed oral disorders. It was concluded that hard foods with sharp edges such as maize straw as well as acidic foods such as silages, favor the development of these disorders. Furthermore, the study shows that the use of mineral supplementation prevents the appearance of oral lesions.

**Abstract:**

Oral disorders constitute a significant cause of weight loss in sheep. In a study of disorders of the oral cavity of 36,033 sheep from 60 meat sheep flocks in Spain, we looked for management risk factors associated with chronic oral lesions. Mandibular and maxillary disorders were assessed as an external manifestation of oral lesions by palpation, searching for tissue swellings, fistulae, or open wounds. The prevalence of flocks containing sheep with jaw disorders was 98.3%, with an average individual prevalence of 5.5%. The majority of lesions were located in in the mid-region of the mandible, and the most relevant risk factor was increasing age. Use of acidic diets based on silage and inclusion of hard foods, such as maize straw or hay, was associated with the occurrence of jaw inflammation. It was concluded that hard diets containing plant material with edges and sharp areas, plus acidic foods including silage, are more likely to cause lesions of the gingiva, enabling entry of pathogens to the oral mucosa that eventually establish locally in bony tissues, usually as mandibular osteomyelitis. It was also observed that mineral supplementation appears to prevent the occurrence of these lesions.

## 1. Introduction

Efficient digestion by ruminants requires healthy oral tissues for prehension and mastication, with oral lesions recognized globally as a significant cause of ovine ill-health. Although there are many causes of chronic weight loss in sheep, lesions of the oral cavity should always be considered when examining animals of low body condition score. Oral lesions can affect an individual or the herd and can be a relevant cause of culling [1,2,3].

There are a wide variety of disorders that can affect the oral cavity. Stomatitis is a process that affects the oral mucosa and is normally of infectious origin. Affected animals will experience weight loss and, depending on the etiology, the animal will show other typical symptoms of the disease [4]. Likewise, dental disorders are many and very frequent in sheep and all of them reduce their feeding efficiency, especially in grazing, causing weight loss [1]. Different percentages of dental disorders ranging from 8 to 48% have been referred to in several studies [5,6,7]. Malocclusion is another common disorder that may be the consequence of a congenital malformation, such as prognathism or brachignatia, or acquired as a consequence of the mismatch of the teeth [8]. However, this condition is more serious when it affects the molars. Excessive or irregular wear of premolar and molar teeth leads to poor chewing of fibrous food and subsequent loss of weight [9]. Similarly, the wear of the incisors is a prevalent condition in sheep. Excessive incisor wear has been related to feeding characteristics, with the consumption of very hard and fibrous grasses, or with defects in the enamel [10,11]. In general, excessive wear causes a progressive thinning of the affected animals, since it reduces their ability to graze. In Angora goats, a 30% wear on the permanent incisors was associated with a 20% decrease in mohair production and a 7.5% reduction in fiber length [12]. The “broken mouth” is an oral disorder that involves lengthening of the incisors due to retraction of the gum, mobility, and premature loss. Loss of incisors is frequent with age, but premature loss constitutes a severe problem because it causes an early elimination of sheep, since they are not able to graze appropriately, especially when the grass is short and hard [1]. Studies based on slaughterhouse data carried out in Great Britain indicate that the loss of incisors is observed in 60–70% of the animals that go to slaughter [13]. Genetic selection, adequate supplementation in times of food shortage, and supplementation of calcium, phosphorus, and vitamin D, especially calcium, favor a better state of the bone and greater firmness and permanence of the incisors [14].

Finally, periodontal disease is one of the most severe diseases that affect the sheep mouth, mainly due to the consequences that it can have. It is a polymicrobial infectious disease that damages the supporting tissue of the teeth and causes occlusion change, tooth loss, and difficulty in rumination. Its origin is multifactorial, and it has to do with the bacterial flora of the mouth, the immune response of the individual, as well as with certain metabolic and environmental factors [2,15,16,17,18]. The prevalence is usually high in herds. In cattle, when 10.00% of the animals show a swollen face, the prevalence can reach up to 60.00% of the herd [19]. In sheep, after external examination of the jaw of 545 individuals, alteration or increase in mandibular size was detected in 3.70% of animals. In the post-mortem examination of 39 young animal heads, the presence of macroscopic lesions was determined in 51.30% of them [20]. In another slaughterhouse study, 227 of 1465 culled sheep heads analyzed (15.5%) had evidence of mandibular osteomyelitis that was externally palpable, with an increase in thickness in the area of the affected body [3]. In sheep, periodontal disease can begin at an early age with gingivitis, which becomes chronic and causes pain while chewing. The accumulation of food debris around the tooth, below the level of the gum, favors the progress of the infection in-depth, eventually causing mandibular inflammation. Sheep that suffer this type of injury can be young and, despite having a proper diet, lose weight due to pain and difficulty chewing fibrous food [1,2,7]. Animals affected by periodontal disease have an inflammatory reaction not only from the gum but also from the base of the tooth and, in severe cases, from the surrounding bone tissue. Those most affected presented a swelling of the submaxillary and parotid lymph nodes, loss of molars, signs of pain on palpation and chewing, deterioration of body condition, and animal welfare. In the most severe cases, the presence of abscesses and fistulas was recorded [21]. These animals reached a state of extreme emaciation and could not stand upright. The introduction of bacteria through unnatural openings causes the infection to progress in-depth and causes pulp and alveolar lesions, affecting the bone that surrounds the tooth, which can lead to deformation of the bone, since inflammation of the medullary cavity invariably affects nearby bone tissue. In the literature, there are several ways to name this disease: “lumpy jaw”, swollen jaw, osteomyelitis of the jaw, or disease of the jaw. The term actinomycosis is also used by extension because this process has traditionally been related to the presence of *Actinomyces bovis* in cattle, although other implicated microorganisms, such as *Staphylococcus aureus* or *Corynebacterium* sp., often appear [22].

Mandibular disorders in sheep become visible only when the animal loses too much body weight or when it stops eating due to the inability to chew food. In advanced stages, it is also possible to detect, after a proper examination, inflammation in the jaw area. For the diagnosis of these disorders, it is necessary to perform external palpation of the jaw. In this case, a hard bulge can be seen in the molar region and, in some cases, the presence of a fistula on the ventral or lateral aspect of the mandible [1]. The differential diagnosis of mandibular disorders, in addition to inflammatory processes, should consider neoplasia, although very few cases of jaw tumors have been described in ruminants [23,24,25].

An oral examination should be performed at least annually in sheep flocks. The routine culling of animals with oral lesions likely to compromise their future health, welfare, and production is required. Oral lesions in otherwise-healthy breeding ewes culled before the end of their natural reproductive life are a major cause of increased flock replacement costs [21,26,27]. Examination of the incisor teeth of sheep by the manual reflection of the upper lips is generally a simple and straightforward procedure in adequately restrained animals. However, a thorough evaluation of the premolar and molar teeth is challenging, with abnormalities of these teeth often going unnoticed until inflammation of the bone occurs, or lesions are identified during post-mortem examination [1,9]. It is likely that the difficulty of examining the entirety of the mouth in live sheep has resulted in there being very few studies that have analyzed in depth the mandibular and maxillary lesions in these animals. An investigation of the distribution and prevalence of mandibular osteomyelitis and lumpy jaw in wild sheep in North American and Eurasia concluded that lumpy jaw was rare in Eurasian wild sheep, with prevalence ranging from 0.0 to 7.1%, although in domesticated breeds the average prevalence was 5% [28]. However, this disorder was found widespread in North America, with a prevalence ranging from 23.3 to 29.3% in wild sheep [28]. A high prevalence of mandibular inflammation was also reported in sheep, with 15% of domestic ewes affected [29]. Furthermore, outbreaks of mandibular osteomyelitis caused by *Pseudomona aeruginosa* have been reported in Brazil, Iran, and Spain, with prevalences of 12.0% [30], 20.0% [31], and 29.2% [32].

In order to improve the knowledge of disorders of the oral cavity of sheep, we looked for the presence of mandibular and maxillary disorders in a large cohort of Spanish sheep. The aim of this study was to establish the prevalence of jaw inflammation and identify the possible farm risk factors that predispose to the occurrence of these lesions in sheep.

## 2. Materials and Methods

All procedures were carried out under Project License PI 11/16 approved by the Ethics Committee for Animal Experiments from the University of Zaragoza. The care and use of animals were performed in accordance with the Spanish Policy for Animal Protection RD53/2013, which meets the European Union Directive 2010/63 on the protection of animals used for experimental and other scientific purposes.

A total of 36,033 sheep belonging to 60 meat sheep flocks located in the Aragon region of the northeast area of Spain, were studied over two years (2016 and 2017). Each sheep was examined individually by the same veterinarian, with detailed clinical examination of the mandible and maxilla performed by external palpation, searching for tissue swellings, fistulae, or open wounds. Evidence of presumptive inflammation in the jaw was palpated as an increase in tissue thickness in the vicinity of affected mandibular or maxillary bones.

During the clinical examination, mandible and maxilla and also left and right mandibular bodies (corpus mandibulae) were examined, demarcating the abnormal areas and recording the location of the lesion as either in the anterior, middle, or posterior zone of the mandible or maxilla. This information was collected in clinical files created for the study, including the individual electronic identification number of each animal, plus the breed, gender, and age. The age of the animals was obtained from the electronic identification that indicated the year of birth.

Possible risk factors influencing the incidence of mandibular and maxillary osteomyelitis in sheep were collected from each farm: flock size, breed, production system, grazing system, diet, mineral supplementation, and cleaning and disinfection. Farm and management factors were initially analyzed individually to determine the influence that each factor had on the prevalence of jaw osteomyelitis. The collective data was then examined by binary logistic regression.

Flocks were classified according to the management system as intensive (n = 3) and semi-intensive (n = 57), by following the criteria of the European Food Safety Authority [33]. In the Aragón region, semi-intensive production systems are based on stabling the animals at the end of gestation and during lactation (1.5–2 months), remaining grazing the rest of the year. Sheep managed by intensive production systems were permanently kept indoors and fed with total mixed ration. Aragon is a dry area, where average annual rainfall does not exceed 350 mm. However, due to the proximity of the Pyrenees mountains and the presence of abundant water reservoirs, there are many irrigated farming areas where alfalfa and maize are mainly cropped. Sheep flocks managed in the semi-intensive system are fed the wastes of the crops in irrigated and non-irrigated areas, where cereals are predominantly grown. Data on the type of grazing carried out by the flocks and food supply during stabling periods were also collected. Therefore, the flocks were divided into those which were kept indoors and fed with total mixed ration (n = 3), those that grazed only in rainfed areas (n = 14), those that grazed only in irrigated areas (n = 14), and those that did mixed grazing in both types of fields, rainfed and irrigated (n = 29). As mineral supplementation is a common practice in sheep farms, the relationship between mineral intake and oral lesions was also analyzed, as was the cleaning and disinfection of the facilities.

In the statistical study, frequency was used to define the lesion profile and individual prevalence according to different risk factors. In addition, association techniques between variables were applied, including the chi-squared test, to reveal relationships between qualitative variables. In this case, the Fisher correction was applied in the event that some of the values collected in the boxes were less than five animals. The risk was also calculated whenever possible (tables 2 × 2). Finally, a binary logistic regression was conducted to determine the factors involved in the presentation of mandibular osteomyelitis. This regression was performed considering as categorical all the qualitative variables of the study and taking as reference the first of the categories of each of them. The “forward conditional” method was applied relating significance to the input of 0.05 and for the output of the model of 0.10. SPSS (IBM Corp. released in 2011. IBM SPSS Statistics for Windows, Version 20.0. Armonk, NY: IBM Corp.) and Microsoft Excel (11. Corporation Microsoft, Redmond, WA, USA) were used for statistical analysis. Statistical significance was inferred when *p* < 0.05.

## 3. Results

Of the 36,033 animals examined, 34,870 were females (96.8%) and 1163 were males (3.2%). The average age of the studied animals was 4.8 years, with a range from less than one year to 17 years, although for the statistical study, animals older than 12 years were grouped as one cohort. The prevalence of flocks containing sheep with oral lesions was 98.3% (59 out of 60 farms), with an average individual prevalence of 5.5% (1972 out of 36,033 animals), although considerable variability between flocks was observed (0.9 to 13.9%). Furthermore, 1734 of those 1972 affected animals had a single lesion (87.9%), and 238 had at least two lesions (12.1%), either in different mandibular bodies, right and left, or in different arches, maxilla or mandible. Lesions in the mandible (97.8%) were much more frequent than in the maxilla (2.2%), with similar proportions on both sides (46.8% right vs. 53.2% left) of the jaw. Inflammations were generally located in the mid-region of the jawbones (71.8%), and most lesions were closed, without fistulae (98.3%).

The average size of the flocks was 600 animals, with a range from 47 to 2339. The most common breed was Rasa Aragonesa, a local meat breed, although other prolific breeds and crossbred sheep were also present in the farms. In the study, 81.4% of the analyzed sheep had access to minerals, and 18.6% did not. Furthermore, 36.9% consumed mineral blocks, and 44.5% consumed only natural salt stones.

**Figure 1:**
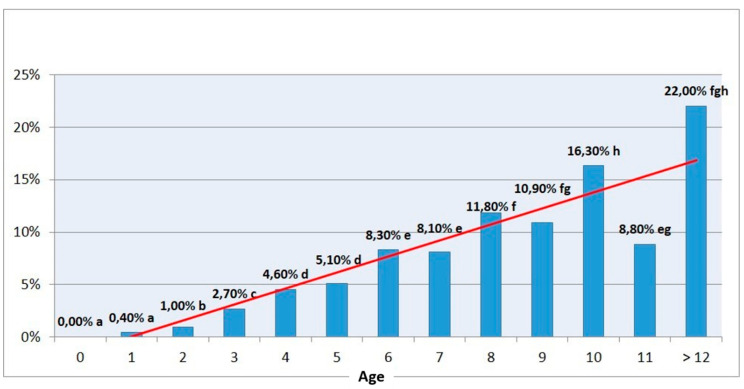
Percentage of mandibular lesions according to the age of the affected sheep at the time of the study. The age groups with different letters (a, b, c, d, e, f, g & h) show statistically significant differences (*p* < 0.05) between them. The line represents the trend line of the percentage of mandibular lesions in relation to age (an increase in the percentage of lesions is observed as the age of the animal increases).

The most relevant farm risk factor influencing the incidence of mandibular and maxillary osteomyelitis in sheep was increasing age (*p* < 0.001) (Figure 1). A 10-year-old animal had a 46 times greater risk of having jaw osteomyelitis than a one-year-old animal and an almost 20 times higher risk than those at two years of age, although these results varied considerably depending on the flock, suggesting that other factors were essential to determine the presence of the disease in the farms. Ewes presented more mandibular and maxillary osteomyelitis than males (5.5% vs. 4.2%); however, this difference was not significant (*p* = 0.055). In addition, the average age of males was lower than females (3.7 vs. 4.8). No differences between the different breeds were observed.

**Figure 2:**
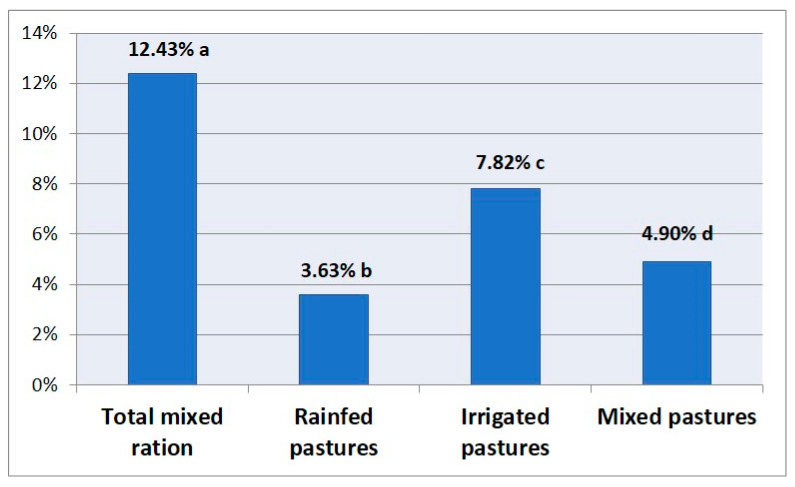
Average collective prevalence of mandibular osteomyelitis according to the feeding system. Different letters mean statistically significant differences (*p* < 0.05).

Significant differences were identified following an assessment of the influence of the production system on the presence of mandibular osteomyelitis (12.4% in intensive vs. 4.9% in semi-intensive, *p* < 0.001). The risk of animals suffering these lesions was 2.7 times higher on intensive than on semi-intensive farms. Flocks grazing only on rainfed pastures had a lower presence of mandibular lesion than those that exclusively grazed on irrigated fields (3.6% vs. 7.8%, *p* < 0.001) (Figure 2). In general, it was concluded that grazing feeding had a protective effect in comparison with feeding in permanent stabling (4.9% vs. 12.4%; *p* < 0.001). Specifically, rainfed, irrigated, and mixed types had 4, 2, and 3 times less risk, respectively, of presenting jaw inflammation than those in permanent stabling.

Use of acidic diets based on silage was also associated with the occurrence of jaw inflammation (8.5% vs. 4.9%), causing an almost two times greater risk of an animal suffering a mandibular lesion than those which did not consume it (*p* < 0.001). However, dehydrated alfalfa pellets appeared to have a protective effect and animals that were fed dehydrated alfalfa had 1.5 times less risk of suffering the oral pathology. The percentage of animals with jaw swelling when fed dehydrated alfalfa pellets was 4.9%, compared to 7.1% for those animals that did not have access to this feed (*p* < 0.001). Furthermore, the inclusion of hay in the diet was also associated with the occurrence of osteomyelitis (6.9% vs. 4.8%; *p* < 0.001), resulting in the risk of suffering oral lesions being 1.5 times greater in these flocks. However, despite maize straw being a hard and sharp-edged product, when consumption of maize straw was analyzed individually, no significant differences were found (*p* = 0.221), although those who consumed maize straw had a higher percentage of jaw swelling (5.6% vs. 5.3%).

**Figure 3:**
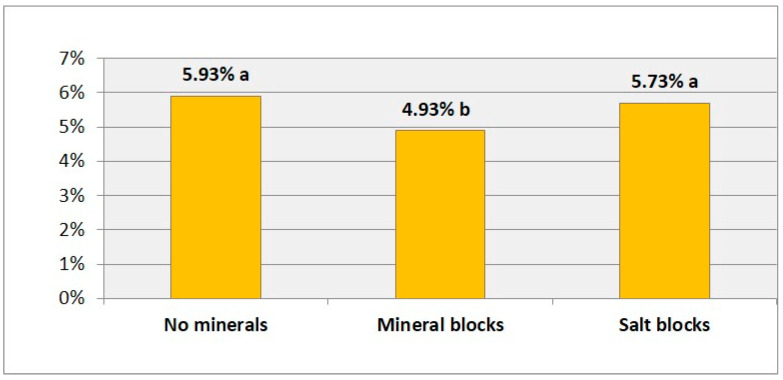
Average collective prevalence of mandibular lesions according to mineral supplementation and type of minerals used. Different letters mean statistically significant differences between the groups (*p* < 0.05).

The flocks receiving dispensed mineral blocks had a lower prevalence of oral lesions compared to those consuming natural salt stones (4.9% vs. 5.7%, *p* = 0.002) and those that did not consume any type of mineral (5.9%, *p* = 0.003). However, no statistical differences were found between those flocks using natural salt stone and those without mineral supplementation (Figure 3). Thus, the use of mineral blocks provided protection that was 1.2 times more than not providing the blocks (*p* = 0.003) or allowing consumption of salt stones.

**Figure 4:**
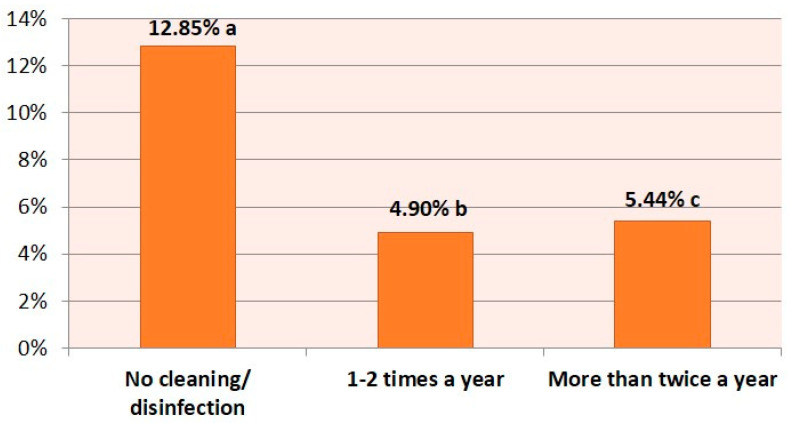
Average collective prevalence of jaw damage depending on the cleaning and disinfection of the facilities and the frequency thereof. Different letters mean statistically significant differences between the groups (*p* < 0.05).

Observations on cleaning and disinfection confirmed that those farmers that did not clean and disinfect their facilities between the different lambing periods had the highest percentage of mandibular lesions (12.8%, *p* < 0.001) (Figure 4).

Finally, when all the farm risk factors were analyzed by binary logistic regression, the model obtained (Nagelkerke R2 = 0.295) showed that factors which influence the incidence of oral lesions were interrelated (Table 1). The analysis demonstrated that the occurrence of mandibular lesions was associated with animal age and type of feed supplied. Contrary to the individual analyses, when analyzed by logistic regression, the inclusion of maize straw in the diet was of significance in the presence of mandibular osteomyelitis. Interestingly, the animals that were fed throughout the year in irrigated fields displayed a higher incidence of mandibular lesions.

It was concluded that hard diets containing plant material with edges and sharp areas, plus acidic foods including silage, are more likely to cause lesions of the gingiva, enabling entry of pathogens to the oral mucosa that eventually establish locally in bony tissues, mainly as mandibular osteomyelitis. It was also observed that mineral supplementation to the animals appears to prevent the occurrence of these lesions.

## 4. Discussion

Few studies have been carried out analyzing the prevalence and risk factors that influence the presence of oral lesions, although this disorder is an important cause of economic loss and early culling in ovine production [3]. The individual prevalence obtained in our study (5.5%) was similar to that obtained by other authors in a study carried out on European domestic sheep (5%) [28]. Although higher individual prevalences of oral lesions have been described in the literature, these are always associated with outbreaks related to the entry of a highly pathogenic microorganism onto the farm [29,30,31,32].

The location of a lesion that occurs when the bones of the oral cavity are infected has been well defined in this work, and it is in full accordance with the work previously carried out by this team in the mandibular lesions of culled sheep at the abattoir [3]. The lesion appears mainly in the central region of the mandible and in equal proportion on both sides. The location of the lesion primarily on the lower arch or mandible may be due to the effect of gravity, that causes the materials to be more impacted in the lower arch, and worse pus drainage, but also to the different composition of both bones. Whilst the maxilla is spongiform, the mandible has a compact bone structure which is much harder and less penetrable, although vascularization and thus defense mechanisms are less [34]. The entry of infection to the mandible usually results in necrosis, with attempts to wall-off inflammatory tissues that may fail, occasionally leading to exudation of necropurulent content and generation of fistulae [34].

As cited by other authors, the most relevant farm risk factor influencing the incidence of maxillary and mandibular osteomyelitis in our study was increasing age. In an outbreak of periodontitis in sheep in Brazil, affected animals were noted to be generally older than 36 months [21]. However, in Iraq, it was concluded that oral lesions were more common in the youngest animals (1.5 to 2 years), with a decrease in the percentage of affected animals as the age increased [35]. In the present study, as the sheep get older, the more the exposure to the main risk factors, such as harsh and acidic diets, the more likely they are to suffer from these lesions.

As observed in wild sheep [28], in our study ewes presented mandibular lesions more frequently than males, although these differences were not significant and this probably reflected that the average age of males was lower than females (3.7 vs. 4.8). When males and females of the same age were analyzed together, no differences between genders were obtained. In the wild sheep, the authors associated this observation with different feeding strategies between females and males, with the latter cohort not taking care for the survival of offspring and able to search for better food [28].

Different surveys have discussed the influence of breed on the presence of jaw disorders [26,35]. In Iraq, it was concluded that oral lesions were more common in the Awassi breed (82.7%) than in the Karadi (12.9%) and Hamdani (4.4%) breeds, and that these differences were statistically significant [35]. An association of oral lesions with the breed was not found in our study.

Of relevance to the sheep production system, this study identified that the risk of suffering mandibular osteomyelitis was higher in intensive than in semi-intensive systems. These findings are in concordance with a study from Iraq, where a difference in the prevalence of oral lesions between animals reared outdoors (10.9%) and those reared indoors (89.0%) was identified [35]. Likewise, in our study, it was concluded that hard diets containing plant material with edges and sharp areas, plus acidic foods, including silage, are more likely to cause oral lesions. Silage consumption has previously been noted to cause a severe problem of dental decay in animals selected for replacement [36]. Decay and damage to the tooth enamel caused by silage can promote the impaction of food and the overgrowth of teeth, causing damage to adjacent structures [27].

Similarly, other authors have suggested that fibrous food can damage the gums, causing wounds [21]. In a flock in Brazil fed in the field with *Panicum maximum* or Massai grass and supplemented at home with elephant grass (*Pennisetum purpureum*), 3.7% of animals were diagnosed with mandibular inflammation at one month following commencement of this diet [21]. Lesions of the oral cavity mucosa and the subsequent osteomyelitis have also been associated with the abrasions caused by rough forage and thorn bushes during grazing [31]. In addition, it was seen in our work that the animals that were fed throughout the year in irrigated fields displayed a higher incidence of mandibular lesions. This may reflect that the main crops planted in these fields are alfalfa and maize. The maize crops wastes are likely to produce similar effects on the gingiva of sheep to those that are produced by access to maize straw during the periods of stabling (it is essentially the same product although packaged).

This study observed a protective effect of mineral supplementation in reducing oral pathology. Similarly, it was concluded in a study that the supplementation of calcium, phosphorus, and vitamin D, especially calcium, favor a better state of the bone and greater firmness and permanence of the incisors [14]. This likely reflects that the composition of the jawbone (enamel, dentine, and cement) includes phosphate, calcium, and other minerals [37]. As the process of mineralization is progressive and discontinuous, particularly for tooth enamel, the state of oral bony structures can vary depending on the composition of the diet, with optimal dietary mineral contributions favoring ossification and greater strength of dentin and other structures, thus assisting in the prevention of entry of pathogenic microorganisms through the mucous membranes [38].

Finally, cleaning and disinfection of the facilities is shown to be a key factor in avoiding the appearance of jaw injuries. A great difference was observed between those that never clean and disinfect (12.85%) and those that performed it one or two times (4.90%) and more than twice (5.44%). Interestingly, a more significant number of lesions were observed in those who clean and disinfect more than twice a year than those who do it once or twice. This is probably due to the fact that when analyzing this parameter individually, it masks other factors of the farm. In fact, in the binary logistic regression, this factor loses relevance.

## 5. Conclusions

Farm management factors, especially diet, seem to have a clear influence on the presence of oral disorders in sheep. Most of the analyzed farms had animals with jaw lesions (98.33%) with a high average individual prevalence (5.5%). The presence of mandibular lesions increased according to the age of the animals analyzed. The main risk factors in the farm that seem to influence the appearance of mandibular lesions in sheep were hard diets containing plant material with edges and sharp areas, plus acidic foods, including silage. These foods are likely to cause lesions of the gingiva, enabling the entry of pathogens to the oral mucosa that eventually settle locally in bony tissues, usually as mandibular osteomyelitis. Mineral supplementation produced a protective effect.

Based on the significant prevalence of mandibular injuries in the flocks analyzed, periodic examinations of the oral cavity should be carried out for early detection of these injuries in order to treat them or avoid associated risk factors.

## Figures and Tables

**Table 1:** Regression coefficients, standard error (SE), statistics parameters (Wald and freedom degree), *p* values, and odds ratio of predictor variables retained in the final regression models fitted to the occurrence of mandibular osteomyelitis in sheep. The odds ratio was calculated for the reference category in each variable. These categories were no use of maize straw, no use of dehydrated alfalfa pellets, no use of mineral supply, and no use of pasture for maize straw, dehydrated alfalfa pellets, mineral supply, and pasture, respectively.

	Coefficients	SE	Wald	df	p	Odds Ratio
Maize straw	0.220	0.068	10.343	1	0.001	1.246
Dehydrated alfalfa pellets	−0.258	0.055	22.039	1	0.000	0.773
Mineral supply			15.692	2	0.000	
Mineral blocks	−0.276	0.07	15.643	1	0.000	0.759
Salt blocks	−0.179	0.073	5.936	1	0.015	0.836
Pastures			342.517	3	0.000	
Rainfed pastures	−1.391	0.085	269.068	1	0.000	0.249
Irrigated pastures	−0.809	0.092	77.764	1	0.000	0.445
Mixed pastures	−1.191	0.085	198.209	1	0.000	0.304
Age	0.265	0.008	1010.107	1	0.000	1.303
Constant	−3.112	0.105	870.456	1	0.000	0.044
